# Supplementary material for: Topological LC-circuits based on microstrips and observation of electromagnetic modes with orbital angular momentum
Source: Nat Commun. 2018 Nov 2;9:4598. doi: 10.1038/s41467-018-07084-2 (PMC6214976; doi:10.1038/s41467-018-07084-2)
Supplement: Supplementary file 2 — Supplementary Information [file 41467_2018_7084_MOESM2_ESM.pdf]

# Supplementary Information for

# “Topological LC-circuits based on microstrips and observation of electromagnetic modes with orbital angular momentum”

**Li et al.**

## Supplementary Figures

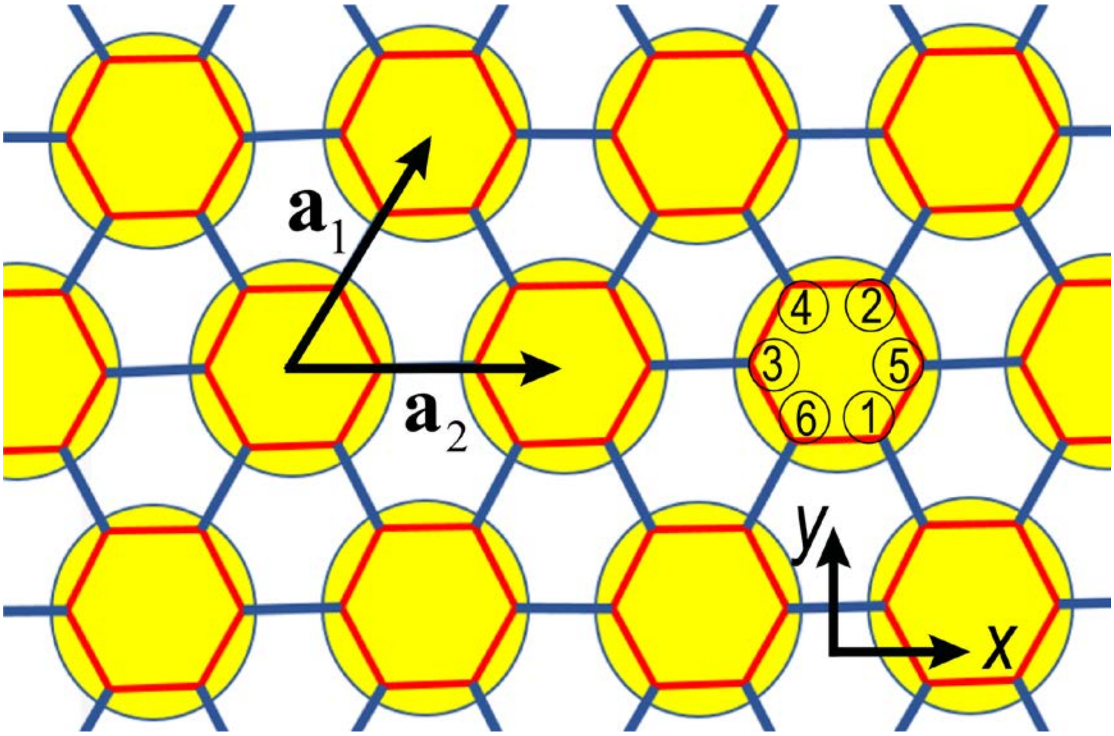


**Supplementary Figure 1** Unit cell with six nodes adopted to describe the LC circuit with on-node capacitors of the uniform capacitance and link inductors of unequal inductances inside (put in red color) and between (put in dark-blue color) unit cells. The nodes are numbered in such a way that the nodes belonging to the two sublattices of the honeycomb structure are grouped separately.

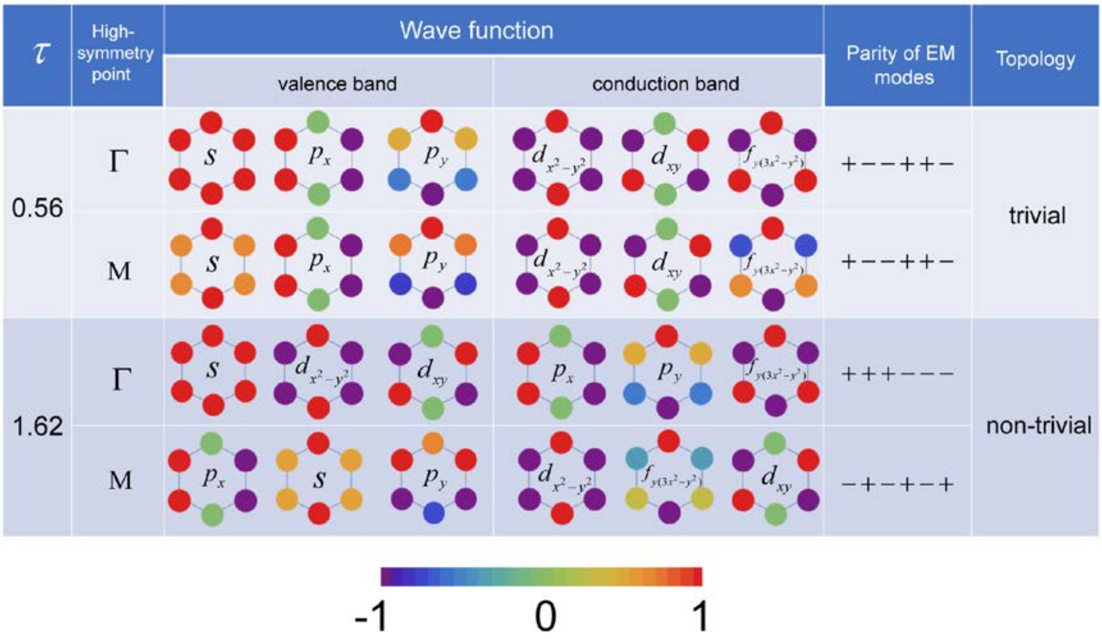


**Supplementary Figure 2** Voltage distributions for the eigen modes and the eigenvalues of the $C_2$ rotation operator denoted by $+$ and $-$ at the Γ point and M point for two typical values of parameter $\tau = L_0/L_1$, obtained by Equation 2 and 3 in the main text.

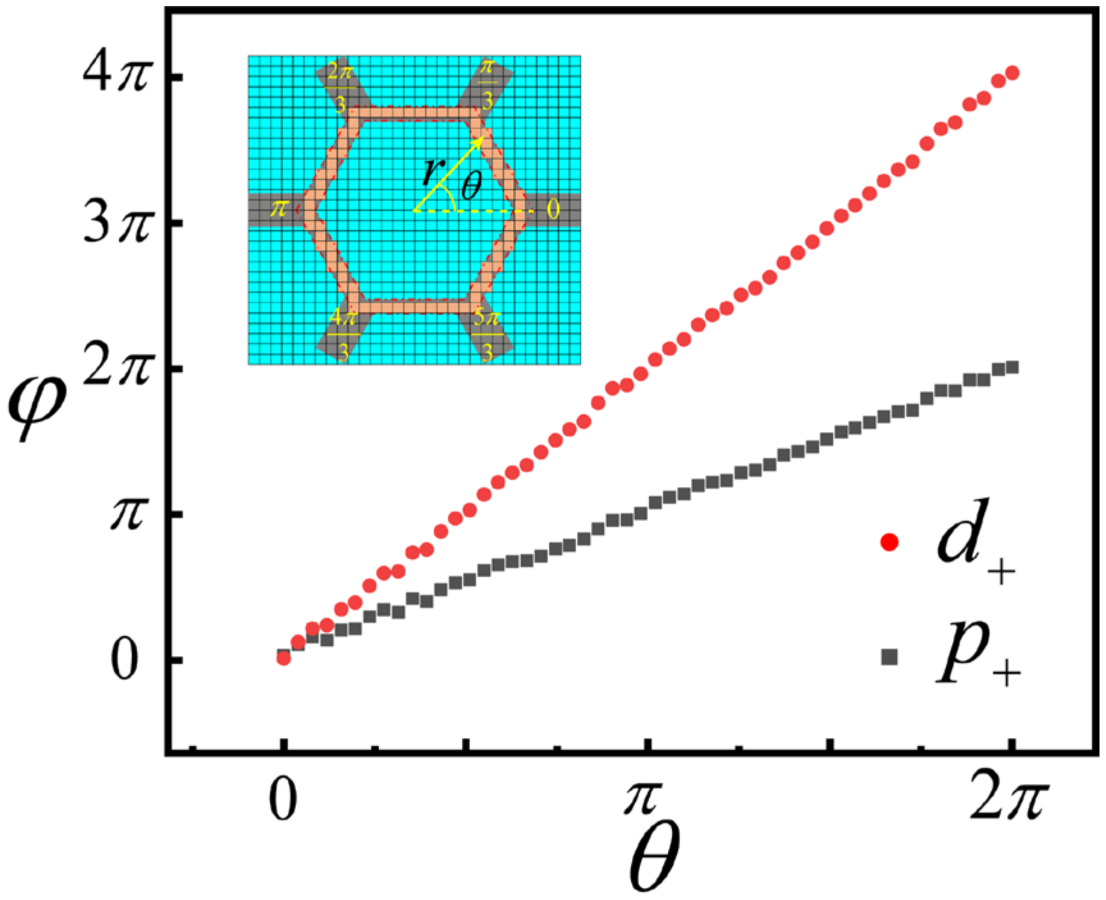


**Supplementary Figure 3** Phase distribution of the eigen wave function of the out-of-plane electric field $E_z$ in a unit cell obtained by the full-wave simulations for the topological sample composed by 14x16 hexagons with the parameters same as those in Figure 3 in the main text. The $p_+/d_+$ eigen wave function is taken at the Γ point and the frequency of 1.59 GHz/1.43 GHz, namely the upper/lower frequency band edge, respectively.

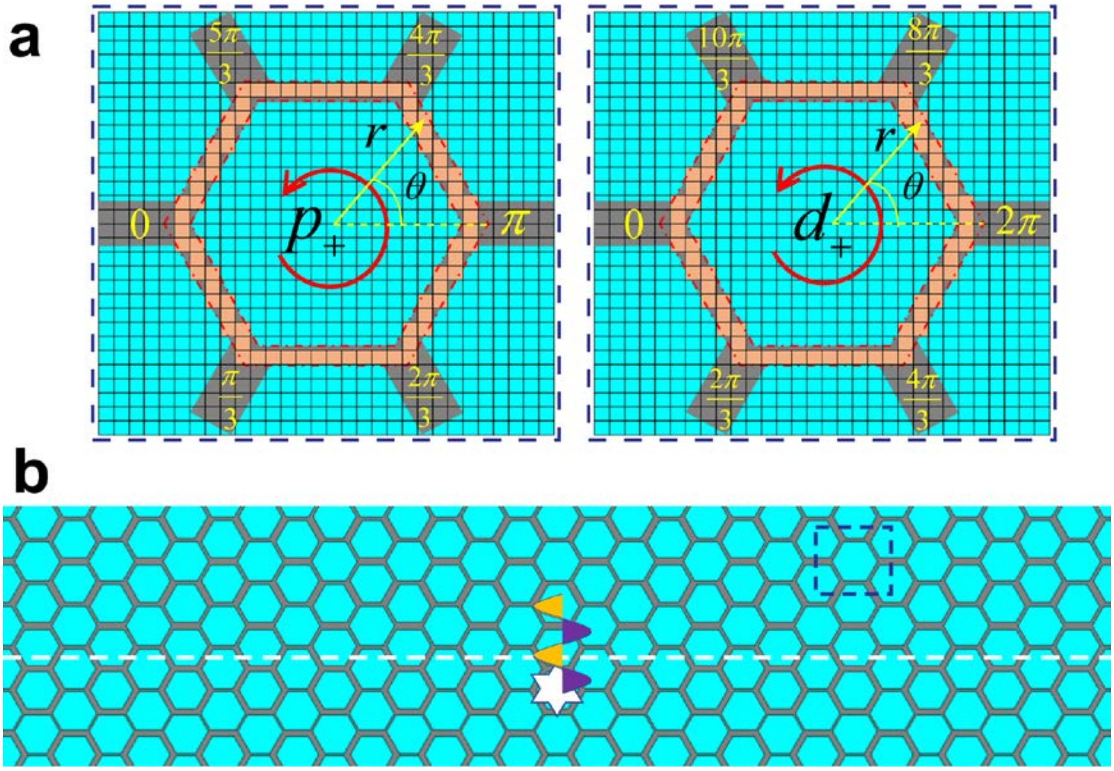


**Supplementary Figure 4** **a**, Schematic of the phase distribution of the eigen wave function of the out-of-plane electric field, which winds $2\pi/4\pi$ in a hexagonal unit cell for $p/d$ orbital. **b**, Schematic of the interface between the topological and trivial microstrip arrays with a source at the center (white hexagonal star), where a typical unit cell bounded by the blue dashed line close to the interface is chosen for analyzing the $p$ and $d$ components in the topological interface modes.

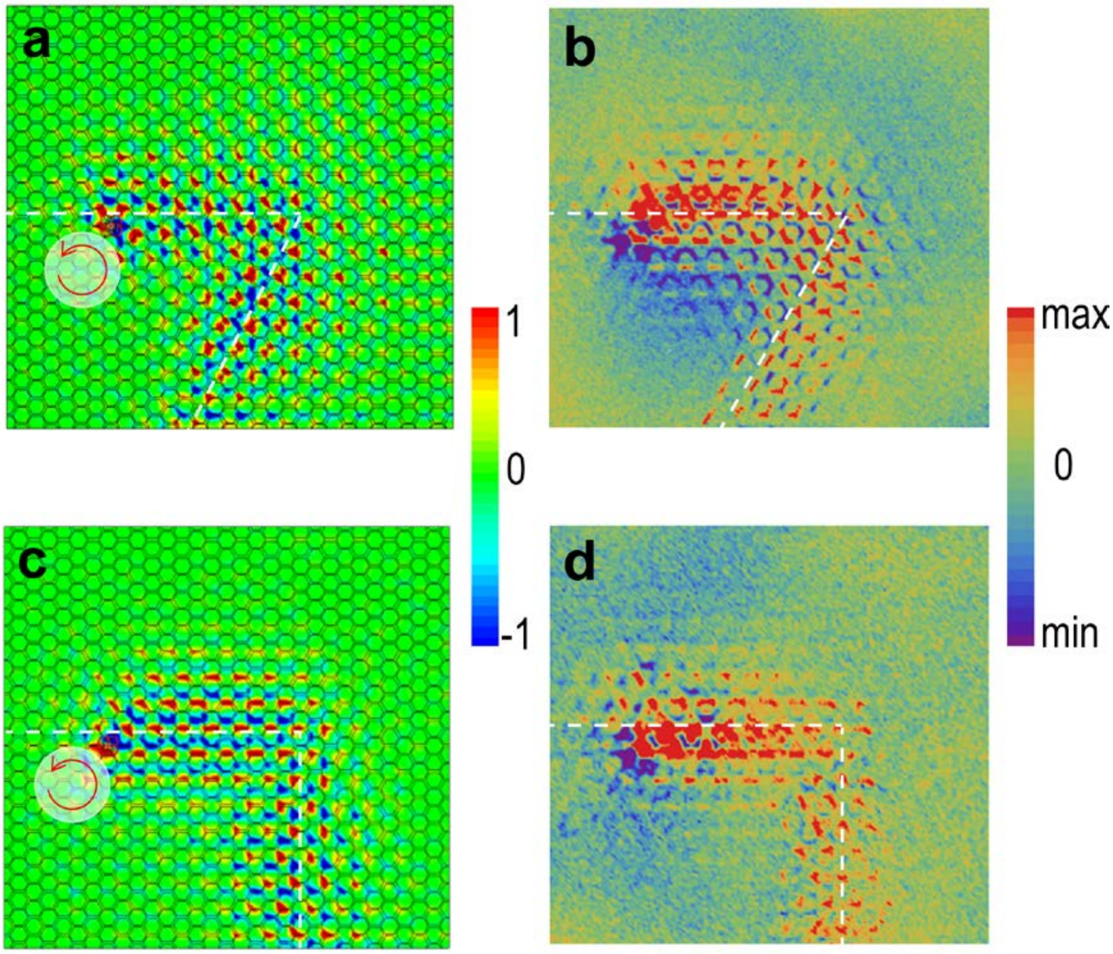


**Supplementary Figure 5 a** and **c,** Distributions of the out-of-plane electric field $E_z$ excited by a pseudospin-up source at 1.47 GHz along the interfaces (white dashed line) with 120$^o$ turn (**a**) and 90$^o$ turn (**c**) obtained by the full-wave simulations. **b** and **d**, Same as **a** and **c** except obtained by experimental measurements at 1.44 GHz.

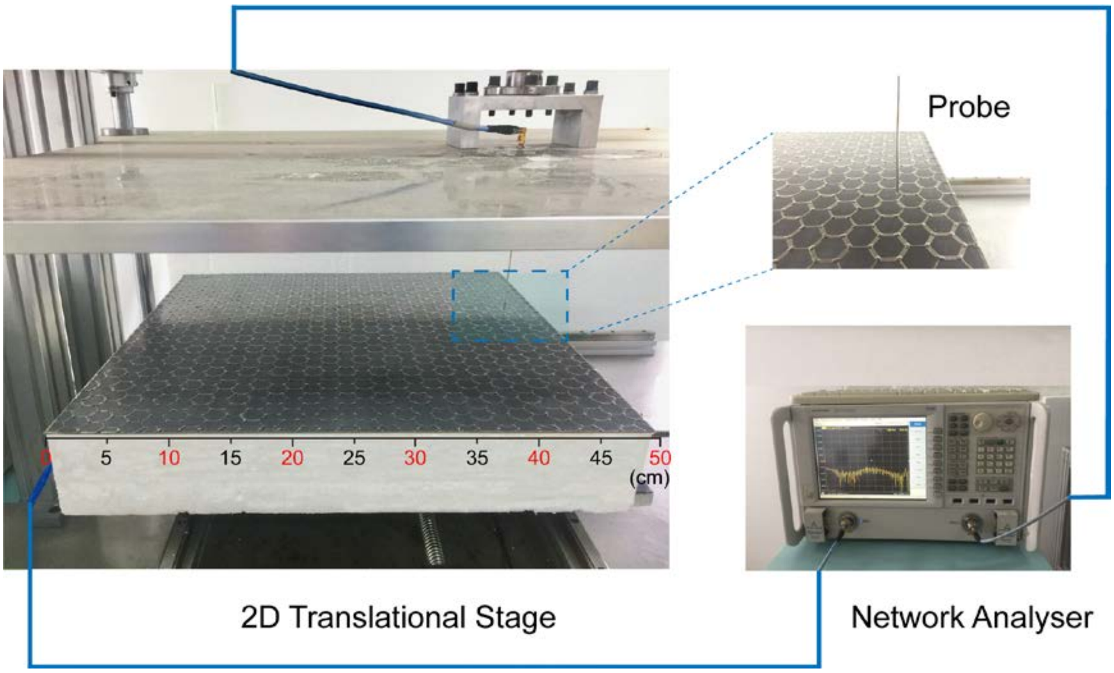


**Supplementary Figure 6** Experimental setup including a 2D translational stage and a vector network analyzer. The vector network analyzer generates EM waves at given frequencies, which are sent to the four-antenna array located in the interface between the topological and trivial microstrip arrays as the source. It also provides the amplitude and phase of out-of-plane electric field $E_z$ based on signals picked up by a small homemade rod antenna mounted in the translational stage above the planar microstrip structure.

# Supplementary Notes

## Supplementary Note 1

Our lumped element circuit is shown schematically in Figure 1**c** in the main text where on-node capacitors with a uniform capacitance $C$ establish shunts to a common ground plane, and link inductors connect the nearest neighbor nodes inside the hexagonal unit cell with inductance $L_0$, and nodes in the next unit cells with inductance $L_1$ (see also Supplementary Figure 1). With Kirckhoff's law from the charge conservation in electrical circuits and voltage drops induced by a current across an inductor $V = L\, dI/dt$ and a capacitor $V = \int I dt/C,$ respectively, the voltage on a given node with respect to the common ground plane is given by

$$V_i = \frac{-1}{C}\int_{t_0}^{t}\sum_{j=1}^{3} I_{ij}\, dt_1 = \frac{-1}{C}\int_{t_0}^{t} dt_1 \sum_{j=1}^{3}\frac{1}{L_{ij}}\int_{t_0}^{t_1}\left(V_i - V_j\right) dt_2, \quad (1)$$

where the system is charge neutralized at a moment $t_0$, and summation is taken over the nearest neighbor nodes. Its differential form serves as the equation of motion for the system

$$d^2V_i/dt^2 = \frac{-1}{C}\sum_{j=1}^{3}\frac{1}{L_{ij}}\left(V_i - V_j\right). \quad (2)$$

Taking the hexagonal unit cell, unit vectors and the numbering of nodes shown in Supplementary Figure 1, the normal frequency modes are described by a vector of six components

$$\mathbf{V} = \mathbf{V}_0 \exp(i\mathbf{k}\cdot\mathbf{r} - i\omega t) \equiv [V_1V_2V_3V_4V_5V_6]^t \exp(i\mathbf{k}\cdot\mathbf{r} - i\omega t), \quad (3)$$

and we obtain the following secular equation

$$\left(2 + \tau - \frac{\omega^2}{\omega_0^2}\right)\mathbf{V}_0 = Q\mathbf{V}_0 \quad (4)$$

where

$$Q = \begin{pmatrix} 0 & Q_k \\ Q_k^\dagger & 0 \end{pmatrix},\ Q_k = \begin{pmatrix} \tau XY^* & 1 & 1 \\ 1 & 1 & \tau X^* \\ 1 & \tau Y & 1 \end{pmatrix} \quad (5)$$

with $X = \exp(i\mathbf{k}\cdot\mathbf{a}_1)$, $Y = \exp(i\mathbf{k}\cdot\mathbf{a}_2)$, $\omega_0^2 = 1/L_0C$ and $\tau = L_0/L_1$, where the asterisk means complex conjugating, as given in the main text.

**Supplementary Note** 2

Considering the band structures in Figure 2 in the main text, a $k \cdot p$ Hamiltonian can be composed around the Γ point based on the four orbitals of $p_\pm$ and $d_\pm$. With the basis $|p_+, d_+, p_-, d_-\rangle$ the 4x4 Hamiltonian is given by[1]

$$H_\Gamma(\mathbf{k}) = \begin{pmatrix} H_+(\mathbf{k}) & 0 \\ 0 & H_-(\mathbf{k}) \end{pmatrix} \tag{6}$$

with

$$H_\pm(\mathbf{k}) = \begin{pmatrix} M + B\mathbf{k}^2 & Ak_\pm \\ A^* k_\mp & -M - B\mathbf{k}^2 \end{pmatrix} \tag{7}$$

where $k_\pm = k_x \pm ik_y$ and $\mathbf{k}^2 = k_x^2 + k_y^2$. The $k \cdot p$ Hamiltonian is block diagonalized into two 2x2 matrices for $p_+/d_+$ and $p_-/d_-$ since the $p_+$ and $d_+$ EM modes, and $p_-$ and $d_-$ ones, carry orbital angular momenta (OAM) different from each other by $\pm \hbar$, which should be linked to each other by finite off-diagonal entries linearly proportional to $k_\pm$, whereas those among the two blocks correspond to high-order corrections in $k_\pm$ negligible in the discussions for the topological properties. With real numbers $M$ and $B$ it is easy to see that the two partial Hamiltonians $H_\pm(\boldsymbol{k})$ are time-reversal-symmetric partners to each other. This $k \cdot p$ Hamiltonian takes the same form as the Bernevig-Hughes-Zhang model of quantum spin Hall effect proposed for HgTe quantum wells[2], where the two 2x2 blocks are associated with the electronic spin-up and -down states. Parallelizing these two Hamiltonians, it is clear that the sign of OAM of eigen EM mode in the present photonic system plays the same role as the spin in spin-orbit coupled electronic systems, indicating that the sign of OAM behaves as a pseudospin degree of freedom.

It is clear that $MB > 0$ for $\tau < 1$ (see Figure 2**a** in the main text), whereas $MB < 0$ for $\tau > 1$ (see Figure 2**c** in the main text), with the latter associated with a band inversion between $p$ and $d$ orbitals and thus a topological state. This agrees with the conclusion derived from the analysis based

on the eigenvalue of $C_2$ rotation operator discussed in Supplementary Note 2.

**Supplementary Note** 3

In microstrip arrays investigated in the present work, the EM field distributes over the strips continuously, with the symmetry features of the EM modes and their impacts to the nontrivial topology remaining unchanged from the lumped element LC circuit. As the evidence, the dependences of the phase of out-of-plane electric field $E_z$ on the continuous azimuthal angle for the two typical eigen wave functions are shown in Supplementary Figure 3, which are obtained by the full-wave simulations for the microstrip array with the parameters same as those for the topological microstrip array in Figure 3 in the main text.

It is clear that at the upper (lower) frequency band edge the eigen wave function is given by $E_z = |E_z| \exp(i\varphi) = |E_z| \exp(il\theta)$ with $l = 1$ $(l = 2)$, where $\theta$ is the continuous azimuthal angle. This is in complete agreement with the picture given by the lumped element LC circuit (see Figure 2**d**-2**g** in the main text).

**Supplementary Note** 4

For a $p_+/d_+$ orbital, the phase of the out-of-plane electric field $E_z$ winds $2\pi/4\pi$ in a hexagonal unit cell counterclockwise, as revealed in Supplementary Figure 3 and shown schematically in the left/right panel of Supplementary Figure 4**a**. This feature can be used to resolve the weights of $p$ and $d$ components in the topological interface modes. For this purpose, we take a square bounded by blue dashed line close to the interface as shown in Supplementary Figure 4**b**, divide it into 30× 30 square meshes as shown in Supplementary Figure 4**a**, and collect the phase of the electric field $E_z$ over the yellow-colored square meshes in both full-wave simulations and experimental

measurements. The weights of $p$ and $d$ components are then evaluated by the following Fourier analyses

$$\left|\int e^{i(\varphi-\theta)}drd\theta\right|^2/A_d^2 \quad \text{and} \quad \left|\int e^{i(\varphi-2\theta)}drd\theta\right|^2/A_d^2 \ , \qquad (8)$$

where $\varphi$ is the phase of the electric field $E_z$ in the mesh located at the distance $r$ and azimuthal angle $\theta$ measured from the center of the unit cell, and $A_d$ is the whole area of the metallic strip as bounded by the red dash-dot lines. As inspected by direct counting, the whole area of integrations in Summplementary Equation 8, $A_n$, namely the summation of the yellow-colored square meshes in Supplementary Figure 4**a**, is slightly smaller than that of the metallic strip $A_d$ with $(A_n/A_d)^2 \approx 0.83$. This explains the apparent deviation of $|p|^2 + |d|^2$ from unity as seen in the Figure 3**q** in the main text. We notice that this discrepancy can be reduced by taking finer square meshes in simulations and experiments. As an approximation, the amplitude of the electric field $E_z$ is omitted in Summplementary Equation 8. We have confirmed that including the amplitude in both numerator and denominator only shifts the two curves in Figure 3**q** in the main text downward slightly, with the over-all trend unchanged. Especially, the crossing frequency between the weights of $p$ and $d$ components remains the same value.

It is noticed that, at the interface where the $C_{6v}$ symmetry is broken partially, OAM is not a good quantum number anymore. This corresponds to the continuous variation of the weights of $p_\pm$ and $d_\pm$ components observed in our experiments when the frequency is swept inside the frequency band gap. It is clear, however, that the main features of the OAM carried by the topological interface modes are inherited from the bulk eigen wave functions as a manifestation of the bulk-edge correspondence. Inversely, the measurement of OAM carried by the topological interface modes reveals the bulk topological properties in a clear way.

**Supplementary Note** 5

OAM of a harmonic EM mode defined in the hexagonal unit cell is related intimately to the local Poynting vector as revealed below. In the present microstrip array, the electric field is along the normal direction of the planar structure $\mathbf{E} = E_z\mathbf{z} = |E_z|e^{i\varphi}\mathbf{z}$, and the magnetic field is aligned laterally due to the Faraday relation

$$\mathbf{H} = -\frac{i}{\mu_0\omega}\left(\frac{\partial E_z}{\partial y}\mathbf{x} - \frac{\partial E_z}{\partial x}\mathbf{y}\right). \quad (9)$$

The local Poynting vector is then given by

$$\mathbf{S} = \mathrm{Re}\,[\mathbf{E}\times\mathbf{H}^*]\,/2 = -\frac{1}{2\mu_0\omega}\mathrm{Im}\left[E_z\left(\frac{\partial E_z{}^*}{\partial x}\mathbf{x} + \frac{\partial E_z{}^*}{\partial y}\mathbf{y}\right)\right] = \frac{|E_z|^2}{2\mu_0\omega}\left(\frac{\partial\varphi}{\partial x}\mathbf{x} + \frac{\partial\varphi}{\partial y}\mathbf{y}\right). \quad (10)$$

For the EM modes with fixed OAM such as $p_\pm$ and $d_\pm$ defined in the hexagonal unit cell, the local Poynting vectors circulate around the edges of hexagon. It is obvious that $p_-$ and $d_-$ orbitals accommodate local Poynting vectors circulating clockwise whereas $p_+$ and $d_+$ orbitals accommodate those circulating counterclockwise, which correspond to the two pseudospin polarizations in the present system.

Now we consider explicitly the amount of angular momentum (AM) carried by the local Poynting vector[3] given in Summplementary Equation 10

$$\mathbf{L} = \mathbf{r}\times\mathbf{S}/c^2 = \frac{|E_z|^2}{2\mu\omega c^2}\left(x\frac{\partial\varphi}{\partial y} - y\frac{\partial\varphi}{\partial x}\right)\mathbf{z}\ , \quad (11)$$

which is a well-defined classical quantity. For the eigen EM mode $E_z = |E_z|\exp(i\varphi) = |E_z|\exp(il\theta)$ where $\theta$ is the azimuthal angle and $l = \pm1, \pm2$ (see eigen wave functions in Figure 2**d**-2**g** in the main text and Supplementary Figure 3), one has

$$\mathbf{L} = \frac{|E_z|^2}{2\mu\omega c^2}l\mathbf{z}. \quad (12)$$

Dividing this AM by the energy density[3], one arrives at

$$\frac{\mathbf{L}}{\varepsilon|E_z|^2/2} = \frac{l\hbar\mathbf{z}}{\hbar\omega} \ , \tag{13}$$

namely a quantized OAM $l\hbar$ along the normal of microstrip plane per photon of energy $\hbar\omega$, which is expected from the wave functions in the lumped element circuit and the microstrip array. It is noticed that at the interface between the two regimes of $\tau < 1$ and $\tau > 1$ the $C_{6v}$ symmetry is broken partially and thus the topological interface modes carry non-integer OAM, which has been revealed in our experiments as shown in Figure 3**e**-3**q** in the main text.

While the features discussed above are common for implementations of the scheme to generate topological EM states based on the $C_{6v}$ symmetry and the $p-d$ band inversion, it is worth mentioning that the microstrip array in the present study provides a unique platform where the local Poynting vectors are evaluated from the distribution of the amplitude and phase of the out-of-plane electric field, which can be measured accurately in the microwave experiments owing to the planar structure. The present work reveals unambiguously the role of OAM in governing the topological EM propagation.

**Supplementary Note** 6

We can also excite topological interface modes by a source launching EM waves with given pseudospin polarizations. As shown in Supplementary Figure 5**a** and 5**c** obtained by the full-wave simulations, the topological interface mode propagates only rightward when the source launches a pseudospin-up EM wave, where a four-antenna array with the phase of EM wave decreasing by π/2 clockwise between neighbor antennas induced by delayed lines is adopted[4]. This unidirectional EM propagation is confirmed clearly by experimental measurements as displayed in Supplementary Figure 5**b** and 5**d**. Results in Supplementary Figure 5 indicate clearly that EM propagations with

topological protection are immune to back-scattering at 120° turn and 90° turn.

**Supplementary Note** 7

Signals generated from a vector network analyzer (Agilent PNA Network Analyzer N5222A) are transported into a port located in the sample, which works as the source for the system. In order to launch EM waves with given pseudospin polarizations, a four-antenna array with the phase of EM wave increasing and decreasing by $\pi/2$ clockwise between neighbor antennas induced by delayed lines is adopted. A small homemade rod antenna of 2 mm length is employed to measure the out-of-plane electric field $E_z$ at a constant height of 2 mm above the planar microstrip structure. We make sure by the full-wave simulations that the field distribution thus measured is almost the same as that at the very surface of the microstrip structure. The antenna is mounted to a two-dimensional translational stage to scan the field distribution over the whole system with a step of 2 mm. The measured results are then sent to the vector network analyzer. By analyzing the recorded field values, we obtain the distributions of the amplitude and phase of the out-of-plane electric field $E_z$, which are used for the analysis on the phase winding and weights of $p$ and $d$ components in the topological interface modes.

**Supplementary References**


1. Wu, L. H. & Hu, X. Scheme for achieving a topological photonic crystal by using dielectric material. *Phys. Rev. Lett.* **114**, 223901 (2015).
2. Qi, X. L. & Zhang, S. C. Topological insulators and superconductors. *Rev. Mod. Phys.* **83**, 1057-1110 (2011).
3. Allen, L., Beijersbergen, M. W., Spreeuw, R. J. C. & Woerdman, J. P. Orbital angular

momentum of light and the transformation of Laguerre-Gaussian laser modes, *Phys. Rev. A* **45**, 8185 (1992).

4. Kapitanova, P. V. *et al.* Photonic spin hall effect in hyperbolic metamaterials for polarization-controlled routing of subwavelength modes. *Nat. Commun.* **5**, 3226 (2014).
